# 液晶分子印迹整体柱的制备及其分子识别热力学

**DOI:** 10.3724/SP.J.1123.2021.01017

**Published:** 2021-11-08

**Authors:** Qin WEI, Xiuxiu CHEN, Lihong BAI, Liang ZHAO, Yanping HUANG, Zhaosheng LIU

**Affiliations:** 天津医科大学药学院, 天津市临床药物关键技术重点实验室, 天津 300070; Tianjin Key Laboratory of Technologies Enabling Development of Clinical Therapeutics and Diagnostics (Theranostics), School of Pharmacy, Tianjin Medical University, Tianjin 300070, China; 天津医科大学药学院, 天津市临床药物关键技术重点实验室, 天津 300070; Tianjin Key Laboratory of Technologies Enabling Development of Clinical Therapeutics and Diagnostics (Theranostics), School of Pharmacy, Tianjin Medical University, Tianjin 300070, China; 天津医科大学药学院, 天津市临床药物关键技术重点实验室, 天津 300070; Tianjin Key Laboratory of Technologies Enabling Development of Clinical Therapeutics and Diagnostics (Theranostics), School of Pharmacy, Tianjin Medical University, Tianjin 300070, China; 天津医科大学药学院, 天津市临床药物关键技术重点实验室, 天津 300070; Tianjin Key Laboratory of Technologies Enabling Development of Clinical Therapeutics and Diagnostics (Theranostics), School of Pharmacy, Tianjin Medical University, Tianjin 300070, China; 天津医科大学药学院, 天津市临床药物关键技术重点实验室, 天津 300070; Tianjin Key Laboratory of Technologies Enabling Development of Clinical Therapeutics and Diagnostics (Theranostics), School of Pharmacy, Tianjin Medical University, Tianjin 300070, China; 天津医科大学药学院, 天津市临床药物关键技术重点实验室, 天津 300070; Tianjin Key Laboratory of Technologies Enabling Development of Clinical Therapeutics and Diagnostics (Theranostics), School of Pharmacy, Tianjin Medical University, Tianjin 300070, China

**Keywords:** 分子印迹聚合物, 液晶, 接枝聚合, 萘普生, 分子识别, molecularly imprinted polymers (MIPs), liquid crystalline, graft polymerization, naproxen, molecular recognition

## Abstract

液晶分子印迹聚合物(MIPs)因刚性液晶单体的加入而在超低交联度水平下也能印迹和识别模板分子,有效解决了传统MIPs因高交联度造成的位点包埋、结合容量低、传质慢等问题。尽管液晶MIPs具有如此独特的优势,但却面临着由于交联度的大幅度降低而导致印迹效果下降的问题。为了研究液晶MIPs的结合特性,制备具有良好印迹效果的低交联液晶MIPs,该文通过二次接枝聚合,制备了一系列不同交联度的液晶分子印迹整体柱,用高效液相色谱法研究了聚合参数与印迹整体柱亲和性的关系。实验中选用三羟甲基丙烷三甲基丙烯酸酯(TRIM)为交联剂,以甲苯和十二醇为致孔剂合成整体柱骨架,并在此基础上以(*S*)-萘普生为模板,加入液晶单体4-氰基苯基单环己基乙烯(CPCE)进行二次聚合接枝。实验中系统考察了流动相中乙腈比例及缓冲液pH值对色谱保留的影响,结果发现液晶单体的加入使得MIPs对萘普生保留控制机制由原来的氢键作用变为了疏水作用;通过动态吸附实验得到的突破曲线经前沿分析及对吸附等温线Langmuir、Freundlich和Scatchard分析拟合,发现交联度为15%时液晶MIPs印迹因子最大(3.78)、非均一性最强,且特异性吸附量高于非特异性吸附量。液晶MIPs的计量置换模型(SDM-R)分析表明,液晶印迹整体柱对模板分子的总亲和力(ln *A*=0.645)明显高于其类似物;而从空间匹配程度看,与液晶印迹整体柱空间匹配程度最高的是酮洛芬而非模板分子,但液晶印迹整体柱对酮洛芬的总亲和力(ln *A*=0.242)不及模板分子的一半,表明在本低交联液晶印迹系统中,空间效应不是决定印迹系统识别能力的主要因素。进一步的分离热力学研究发现,低交联液晶印迹柱的|ΔΔ*H*|<*T*|ΔΔ*S*|,而交联度为70%的非液晶MIPs柱的|ΔΔ*H*|>*T*|ΔΔ*S*|,表明液晶MIPs的分离过程是一个熵控制过程,而常规无液晶MIPs的分离过程是一个焓控制过程。上述结果表明,液晶单体的加入改变了MIPs的识别机制,适当的低交联度可显著提高液晶MIPs的识别性能,因此液晶MIPs这些特质有望使其成为新一代的MIPs。

## Abstract (en)

Molecularly imprinted polymers (MIPs) incorporated with liquid crystalline monomers can imprint and recognize templates at a very low level of crosslinking, thus addressing challenges associated with conventional MIPs, such as the embedding of the imprinted sites, low binding capacity, and slow mass transfer due to the high degree of crosslinking. Compared with traditional MIPs, the prepared MIPs have a greater number of easily binding sites, which can effectively overcome the embedding and low utilization of imprinting sites. Simultaneously, with a decrease in the level of chemical crosslinking, the mass transfer of template molecules can be significantly improved. However, the imprinting effect of liquid crystalline MIPs is generally weaker than that of traditional MIPs due to the low degree of crosslinking. Therefore, to obtain liquid crystalline MIPs with a good imprinting effect, a series of low-crosslinked liquid crystalline molecularly imprinted monoliths were prepared by graft polymerization and evaluated by high performance liquid chromatography (HPLC) to systematically determine the relation between the polymerization parameters and the affinity of the resulting liquid crystalline MIPs. In this experiment, trimethylolpropane trimethacrylate (TRIM) was used to synthesize a monolithic column skeleton with toluene and dodecyl alcohol as porogens. (*S*)-Naproxen was used as a template and liquid crystalline monomer 4-(4-cyanophenyl)-cyclohexyl ethylene (CPCE) was added for grafting to synthesize the liquid crystalline MIP monolith. The influence of the acetonitrile content and pH in the mobile phase on the chromatographic retention of the template molecule was investigated. The results showed that the main force of MIP recognizing naproxen changed from hydrogen bonding to hydrophobic interaction by the addition of the liquid crystalline monomer. Frontal analysis and adsorption isotherm fitting, including Langmuir, Freundlich, and Scatchard fitting, showed that when the crosslinking degree was 15%, the liquid crystalline MIPs exhibited the highest imprinting factor and heterogeneity, and the specific adsorption was stronger than non-specific adsorption. By analyzing the stoichiometric displacement model, the total affinity of the MIP monoliths for the template molecules (ln *A*) was determined to be 0.645, significantly higher than that of its analogues, indicating that the liquid crystalline imprinted monolith had a higher total affinity for the template molecule. The spatial matching degree (*nβ*) of the template molecule to the cavity structures of MIPs was also very high, and only inferior to that of ketoprofen. Nevertheless, the ln *A* value of ketoprofen was only 0.242, which indicated that the spatial effect was not the key factor in determining the recognition ability of liquid crystalline imprinting systems. An analysis of the separation thermodynamics revealed that the separation of the liquid crystalline MIPs was an entropy-controlled process, while that of conventional liquid crystalline-free MIPs was an enthalpy-controlled process. Based on the above results, the addition of a liquid crystalline monomer may alter the recognition mechanism of MIPs, and an appropriately low crosslinking degree can significantly improve the recognition performance of liquid crystalline MIPs, paving the way for a new generation of MIPs.

分子印迹技术是以目标分子为模板,在功能单体及交联剂存在下制备对该分子具有特异性识别能力的聚合物的方法,该技术制得的产物即为分子印迹聚合物(molecularly imprinted polymers, MIPs)^[1]^。MIPs可以与印迹分子特异性结合,其在外消旋体、小分子类似物及生物大分子等分离分析方面具有重要的应用^[2,3,4]^。传统的MIPs通常需要高交联度(大约80%~90%)保持聚合物的空间结构,以实现对模板的识别^[5]^。但是这种高交联度形成的聚合物其内部网格致密,导致分子进出印迹空穴受阻,传质变慢,作为高效液相色谱的固定相会带来严重的色谱峰展宽,导致定性定量困难。因此,需要发展新一代的MIPs,以避免为获得对模板分子良好印迹而产生的高交联度依赖。

制备低交联度MIPs的一种新策略是应用液晶单体^[6]^。液晶单体是一类末端具有可极化基团的刚性棒状分子,在MIPs制备时,液晶单体的加入可以替代部分化学交联剂,利用其刚性棒状结构的相互作用,起到固定柔性的聚合物链的作用,可使得制备的MIPs在很低交联度水平下也能够印迹和识别模板。因为用物理交联取代了部分化学交联,由此制备的MIPs与传统的高交联度MIPs相比具有更易结合的位点,有效减少了印迹位点包埋、位点利用率低的困扰,因此具有更高的结合容量。同时,伴随着化学交联水平的降低,模板分子的传质也大为提高。最近,已有基于液晶单体的MIPs在仿生催化剂^[7]^、手性固定相^[8]^、药物释放材料^[9]^及电化学传感器^[10]^等方面的研究,其都显示出不同以往的优势,因此有望成为新一代的MIPs。

尽管液晶MIPs具有独特的优势,但与传统MIPs相比,其印迹效果由于交联度的大幅降低而下降也是不争的事实。因此研究液晶印迹柱的吸附特性以及特异性吸附与非特异性吸附随交联度的变化规律,对设计新型液晶MIPs并提高印迹效果有着至关重要的意义。但已有的工作只是将液晶MIPs应用于不同领域,尚未对其结合特性进行深入的讨论。本研究拟从色谱分离热力学角度出发,在合成液晶MIPs和色谱条件优化后,对其结合位点数及解离常数等进行系统考察,总结其结合特性及规律,为理性设计新一代液晶MIPs打下良好的基础。

药物和个人护理用品(PPCPs)作为一种新的环境污染物广受研究者关注,这是因为未被除去的PPCPs会在环境中不断累积,从而对生态环境及人类健康产生极大危害,(*S*)-萘普生((*S*)-naproxen, 以下简称萘普生或NAP,结构式见图1)就是其中一种常见的PPCPs污染物。由于在环境水体中存在浓度低、极性强且环境样品基质复杂等特点,常规的萃取技术难以实现对PPCPs有效的分离和富集,因此已成为急需破解的技术难题之一。

**图1:**
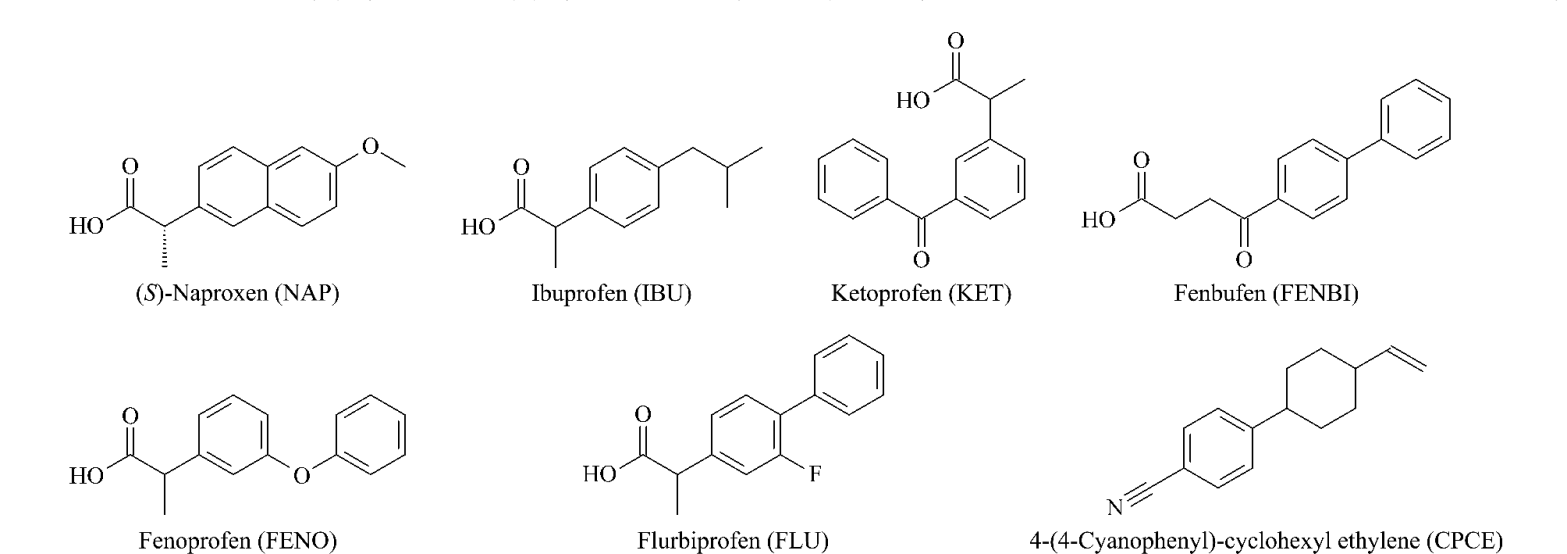
萘普生及其结构类似物和CPCE的结构式

鉴于MIPs的选择性吸附已成为一种有前景的富集水中污染物的方法,在本文实验中我们用三羟甲基丙烷三甲基丙烯酸酯(TRIM)为交联剂,以甲苯和十二醇为致孔剂,在不锈钢管柱中合成整体柱骨架,然后在其上二次聚合接枝,以萘普生为模板,加入液晶单体4-氰基苯基单环己基乙烯(CPCE)合成液晶MIPs整体柱,并进行色谱保留及分离热力学的研究。

## 1 实验部分

### 1.1 仪器及试剂

高效液相色谱仪:CoM 6000(CoMetro Technology,美国);Waters 2487系列(Waters公司,美国)。

以下试剂纯度除特别说明外均为分析纯。萘普生和酮洛芬(ketoprofen, KET)购于浙江仙居化工有限公司,布洛芬(ibuprofen, IBU)、芬布芬(fenbufen, FENBI)、非诺洛芬(fenoprofen, FENO)及氟比洛芬(flurbiprofen, FLU)均购于湖北恒硕化工有限公司,CPCE购于石家庄斯蒂亚诺精细化工有限公司,4-乙烯吡啶(4-VP,色谱纯)、三羟甲基丙烷三甲基丙烯酸酯、二甲基丙烯酸乙二醇酯(EDMA)均购于美国Sigma公司,甲苯(色谱纯)购于廊坊市兴科化工有限公司,异辛烷、十二醇、偶氮二异丁腈(AIBN)均购于天津市科密欧化学试剂有限公司,乙腈(色谱纯)、乙酸(色谱纯)分别购于天津市彪士奇科技发展有限公司、天津市康科德科技有限公司,甲醇(色谱纯)、乙腈、丙酮、乙酸等试剂均购于天津市江天化工技术有限公司。萘普生结构类似物和液晶单体的结构式见图1。

### 1.2 表面接枝印迹整体柱的制备方法

1.2.1 骨架材料的制备

按照表1称取AIBN,加入适量TRIM,再加入致孔剂甲苯和异辛烷或者甲苯和十二醇溶液,超声溶解15 min使之均匀、澄清,通氮气10 min以除去氧气,然后注入不锈钢柱(100 mm×4.6 mm),将两端封住,于48 ℃恒温水浴中反应适当时间。将柱取出并连于CoM 6000 HPLC仪的高压泵上,用乙腈冲洗除去整体柱中的致孔剂,冲洗液总体积约为150 mL。最后用乙腈将系统平衡至基线水平,在流速为0.5 mL/min下测定柱压。

**表1:** 接枝印迹整体柱骨架材料制备的配方

Monolith	AIBN/ mg	TRIM/ μL	Toluene/ μL	Isooctane/ μL	Dodecanol/ μL	Time/ h	Theory plates/ (plates/m)	Back pressure/ kPa
C1	18	1000	1200	1800	0	16	3940	3447.5
C2	18	1000	1200	1800	0	14	9000	703.3
C3	18	1000	1200	1800	0	12	1980	0
C4	18	1000	1200	0	1800	14	1900	2482.2
C5	18	1000	750	0	2250	14	12000	324.1
C6	18	1000	600	0	2400	14	900	0
C7	18	1000	750	0	2250	15	4000	703.3
C8	18	1000	750	0	2250	13	1200	0

AIBN: azobisisobutyronitrile; TRIM: trimethylolpropane trimethacrylate.

1.2.2 接枝MIPs的制备

取模板NAP 0.20 mmol、功能单体4-VP 0.81 mmol和引发剂AIBN 10.8 mg,再按照表2加入CPCE、EDMA及致孔剂甲苯和十二醇,超声溶解20 min,得到均匀、澄清的预聚合液,向其中通入氮气10 min以除去氧气。将骨架整体柱C5连接到CoM 6000高效液相色谱仪上,用同样比例的甲苯和十二醇冲洗,再将预聚合液以0.2 mL/min的速度注入整体柱中,柱两端封住于53 ℃恒温水浴中反应4 h后将柱取出,先用乙腈冲洗以除去整体柱中残留的致孔剂,然后再用甲醇-乙酸(9:1, v/v)混合液冲洗至除去模板分子,流速由0.1 mL/min逐渐增大至1 mL/min,冲洗液总体积约为150 mL。空白印迹柱除不加模板外,其余步骤同上。

**表2:** 接枝到整体柱骨架上的MIPs配方

Monolith	Crosslinking degree/%	CPCE/ mmol	EDMA/ mmol	Toluene/ mL	Dodecanol/ mL
P1	26	2.15	1.05	1.556	0.389
P2	20	2.40	0.80	1.556	0.389
P3	15	2.59	0.60	1.556	0.389
P4	10	2.79	0.40	1.556	0.389
P5	7.5	2.90	0.30	1.556	0.389
P6	5.0	3.00	0.20	1.556	0.389
P7	70	0	1.90	1.556	0.389
P10	30	2.00	1.20	1.945	0

EDMA: ethylene glycol dimethacrylate.

### 1.3 色谱保留考察

将P1柱连接到Waters高效液相色谱仪上,以乙腈-乙酸缓冲盐(50 mmol/L, pH 3.6)为流动相,依次改变乙腈含量为50%~95% (v/v)进行上样。或者以乙腈-乙酸缓冲盐(50 mmol/L, 99:1)为流动相,改变缓冲盐溶液pH(3.0~5.0)进行实验。其中,流动相流速为0.5 mL/min,上样量为20 μL,检测波长254 nm,柱温为28 ℃。获得萘普生及其类似物在不同流动相条件下的保留因子。

### 1.4 动态吸附实验和前沿分析

将印迹整体柱连接到Waters高效液相色谱仪上,用乙腈-乙酸缓冲盐(pH 3.6, 50 mmol/L)(99:1, v/v)作流动相,将不同浓度的萘普生溶液(0.1~0.4 mmol/L)依次上样,以1.0 mL/min的流速流过色谱柱,当流出曲线达到一个稳定的平台,即为完成一个突破曲线。实验中不同交联度的印迹整体柱(P1~P5, P7)都可以获得一系列的浓度对应突破曲线。P6由于无印迹效果无法进行该实验。萘普生的动态平衡吸附量(*Q*)可由公式(1)计算^[11]^:


(1) $Q=A_{0} \frac{V-V_{0}}{v}$

其中*A*_0_为萘普生的浓度,*V*为吸附达到平衡时萘普生的保留体积(可用半高法在突破曲线上测得), *V*_0_为死体积(通过测定丙酮的保留时间算得), *v*为柱床体积。对动态吸附实验得到的突破曲线数据进行前沿分析^[12]^:


(2) $\frac{1}{A_{0}\left(V-V_{0}\right)}=\frac{K_{\mathrm{d}}}{B_{\mathrm{t}} \cdot A_{0}}+\frac{1}{B_{\mathrm{t}}}$

其中*B*_t_为结合位点总数,*K*_d_为解离常数。根据公式(2)绘制1/[*A*_0_(*V-V*_0_)]对1/*A*_0_的关系图。

### 1.5 吸附等温线分析

从突破曲线实验可得不同浓度萘普生在MIPs上的动态平衡吸附量,用热力学模型对其进行拟合并分析。Langmuir方程可以模拟模板分子在印迹固定相表面单分子层吸附达到平衡时*Q*与*C*的关系^[13]^:


(3) $Q=\frac{Q_{0} K_{\mathrm{L}} C}{1+K_{\mathrm{L}} C}$

式中*Q*_0_为单层吸附的最大吸附量,*K*_L_为吸附系数,*C*为达到平衡时分析物在流动相中的浓度。

鉴于印迹固定相为非均匀性吸附材料,我们用Freundlich方程以评估MIPs的非均一性^[13]^:


(4) lg *Q*=lg *K*_F_+1/*n*lg *C*

式中*K*_F_用来表征总结合位点数和平均亲和系数。1/*n*为非均一性指数,1/*n*值越接近0,表明MIPs的非均一性越强,印迹效应越大。

用Scatchard方程对吸附等温线进行拟合,可以评价分子印迹聚合物的结合位点类型。Scatchard方程可写为^[14]^:


(5) $\frac{Q}{C}=\frac{Q_{\max }-Q}{K_{\mathrm{d}}}$

其中*Q*_max_为MIPs最大表观吸附量。根据Scatchard方程,若*Q/C*对*Q*作图呈一良好的直线,则表明MIPs存在一类等价的结合位点。然而有时*Q/C*对*Q*明显呈非线性关系,这表明聚合物的结合位点并不是等价的,但在Scatchard图两端往往具有较好的直线关系,分别代表了高亲和位点和低亲和位点。由Scatchard图两端的直线的斜率和截距可求得两类结合位点的平衡离解常数*K*_d_和最大表观吸附量*Q*_max_。

### 1.6 计量置换研究

将P1柱连接到Waters HPLC仪上,在流速为0.5 mL/min、柱温为28 ℃条件下,将不同量的乙酸作为强氢键竞争性溶剂加入到乙腈中,考察流动相中乙酸含量为0.5%~3.0%(v/v)时对萘普生及其类似物的保留因子的影响。依据计量置换模型(SDM-R),将测得的色谱数据以方程(6)进行拟合^[15]^:


(6) ln *k*=ln *A-nβI*

其中*k*是保留因子,*I*是流动相中的乙酸百分比。ln *A*为溶质与固定相之间总体的亲和力,它包括了溶质与MIPs孔结构之间的空间效应,以及溶质、溶剂和功能单体之间的作用。*β*表示由强溶剂取代的弱溶剂的平衡常数。*n*表示溶质分子吸附在固定相上时,从固定相中释放出的强溶剂分子的数目。

### 1.7 分子识别热力学

为了考察温度对于分离的影响,我们研究了不同温度下模板及其类似物在二次聚合柱(交联度70%、26%、15%、7.5%)上的保留。在流速为0.5 mL/min条件下将温度从25 ℃逐步升到45 ℃,重复进样。根据Van’t Hoff公式^[16]^,利用实验中得到的保留因子*k*和萘普生类似物与萘普生之间的分离因子*α*评估模板及其类似物在MIPs上保留的焓变、熵变和Gibbs自由能。即:


(7) $\ln k=-\frac{\Delta H}{R T}+\frac{\Delta S}{R}+\ln \Phi$

(8) $\ln \alpha=-\frac{\Delta \Delta H}{R T}+\frac{\Delta \Delta S}{R}$

其中,*R*、*T*、*Φ*为分别为气体常数、绝对温度以及相比。

## 2 结果与讨论

### 2.1 表面接枝印迹整体柱的制备考察

由于液晶印迹聚合物具有天然的弹性,难以抵抗HPLC的高压,易变形,难于直接作为HPLC的固定相。应用接枝二次聚合制备的方法可以很好地解决这一问题,即先制备渗透性良好的聚合物整体柱,然后利用表面印迹的方法在整体柱骨架上进行分子印迹。Zhang等^[8]^曾用此思路制备出具有高印迹因子和良好选择性的MIPs并应用于HPLC分离。本研究我们采用类似的方法,选用TRIM为聚合单体,在不锈钢管柱中合成具有良好通透性和固定形态的聚合物整体柱骨架,然后合成接枝印迹整体柱。

2.1.1 骨架材料的制备

骨架材料被合成以后,其表面必须有剩余的双键存在,以保证二次聚合的进行。过去的文献表明,TRIM聚合物中表面双键的剩余量与温度密切相关,即低温聚合会在TRIM聚合物的表面剩余更多的碳碳双键^[17]^。在本实验中,我们选择48 ℃作为反应温度,根据文献^[18]^,在TRIM骨架结构表面约有12%游离的碳碳双键可作为MIPs印迹时的锚定位点。此外,在骨架材料的制备中,为保证聚合物骨架具有较好的通透性以及刚性,我们优化了一系列的聚合参数,包括致孔剂种类和组成、反应时间等。由于使用甲苯和异辛烷为致孔剂时,骨架刚性不够,导致二次接枝无法进行,故我们选择了甲苯和十二醇为致孔剂。根据表1可知C5柱柱效最高,且柱压较小,通透性较好,故最终我们选择其进行后续的实验。

2.1.2 接枝MIPs的制备

对于制备表面印迹的聚合物整体柱来说,尽管各种聚合条件对聚合物的通透性、刚性以及印迹效果都有影响,但本实验决定实验关键成败的却是致孔剂,因为最终制备的聚合物能够承受HPLC的高压是实验的必要条件,因此致孔剂的组成和种类是我们的主要考察因素。目前成功用于制备MIPs薄层的致孔剂体系有:甲苯^[10]^、氯仿^[19]^和一些混合溶剂,如环己醇-十二醇^[20]^、甲苯-异辛烷^[21]^和三元致孔体系(甲苯-异辛烷-DMSO)^[22]^。实验表明,只使用甲苯做致孔剂不能使二次接枝很好地完成,这是因为只用甲苯会导致薄层中小孔结构过多而使柱压增大,导致二次聚合柱无法冲通(P10柱)。而十二醇是致大孔溶剂^[23]^,有报道甲苯-十二醇为很好的致孔剂^[24]^,并且在我们的实验中能够很好地溶解模板NAP和液晶单体。因此,在我们的实验中选用甲苯-十二醇作为MIPs的致孔剂。

2.1.3 接枝印迹整体柱色谱行为及印迹因子

为了系统研究含有液晶单体的低交联度MIPs的结合特性,我们制备了一系列低交联度的MIPs(P1~P6),另外还合成了交联度70%的二次无液晶印迹柱P7与之形成对比(见表2),其中印迹因子可由公式IF=*k*_MIP_/*k*_NIP_计算,*k*_MIP_和*k*_NIP_分别为萘普生在MIPs和对应的NIPs上的保留因子。由表3可看到,随着交联度降低,制得的液晶MIPs模板保留减弱,柱效升高,其中印迹因子在交联度15%时最大,而当交联度为5%时,无印迹效果;相反,当交联度为70%时,即无液晶单体时,制得的MIPs柱效升高,但印迹因子明显低于柱效最低的P1柱。此外,制备的低交联液晶印迹整体柱(P1)能使萘普生与其结构类似物达到基线分离(见图2)。

**图2:**
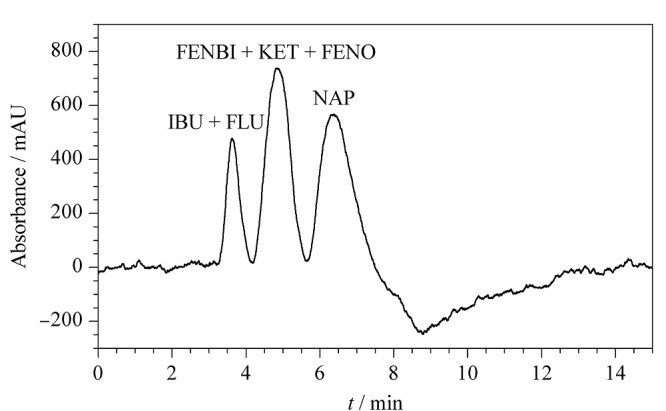
萘普生及其结构类似物在P1柱上的色谱分离图

**表3:** 萘普生在不同印迹整体柱上的色谱参数

Monolith (Crosslinking degree)	t_R_/ min	k	N/ (plates/m)	IF
P1 (26%)	6.70	2.22	2410	2.66
P2 (20%)	6.61	2.18	2880	2.32
P3 (15%)	4.17	1.22	3870	3.78
P4 (10%)	3.71	0.52	3980	1.59
P5 (7.5%)	4.28	0.63	4250	1.44
P6 (5.0%)	4.11	0.50	4900	1.02
P7 (70%)	7.90	1.18	5400	1.66

*t*_R_: retention time of naproxen; *k*: retention factor; *N*: theoretical plate; IF: imprinting factor.

### 2.2 接枝印迹整体柱色谱行为考察

2.2.1 流动相乙腈比例的影响

从图3a可看出,当乙腈含量从50%增加到80%时,萘普生及其结构类似物的保留因子均迅速减小,此时可能是疏水作用主导保留机制;而乙腈含量从80%增加到95%时,萘普生及其类似物的保留因子均稍有增加,此时可能是氢键或其他静电相互作用主导保留机制的结果,这一结果与先前的报道无液晶单体的NAP-MIP研究结果相反^[25]^。因此,这表明由于液晶单体的加入,使得控制印迹系统保留的机制由原来的氢键作用变为了疏水作用。另外,MIPs P1柱对萘普生的保留因子始终大于其他类似物,表明MIPs P1柱具有较好的吸附特异性。

**图3:**
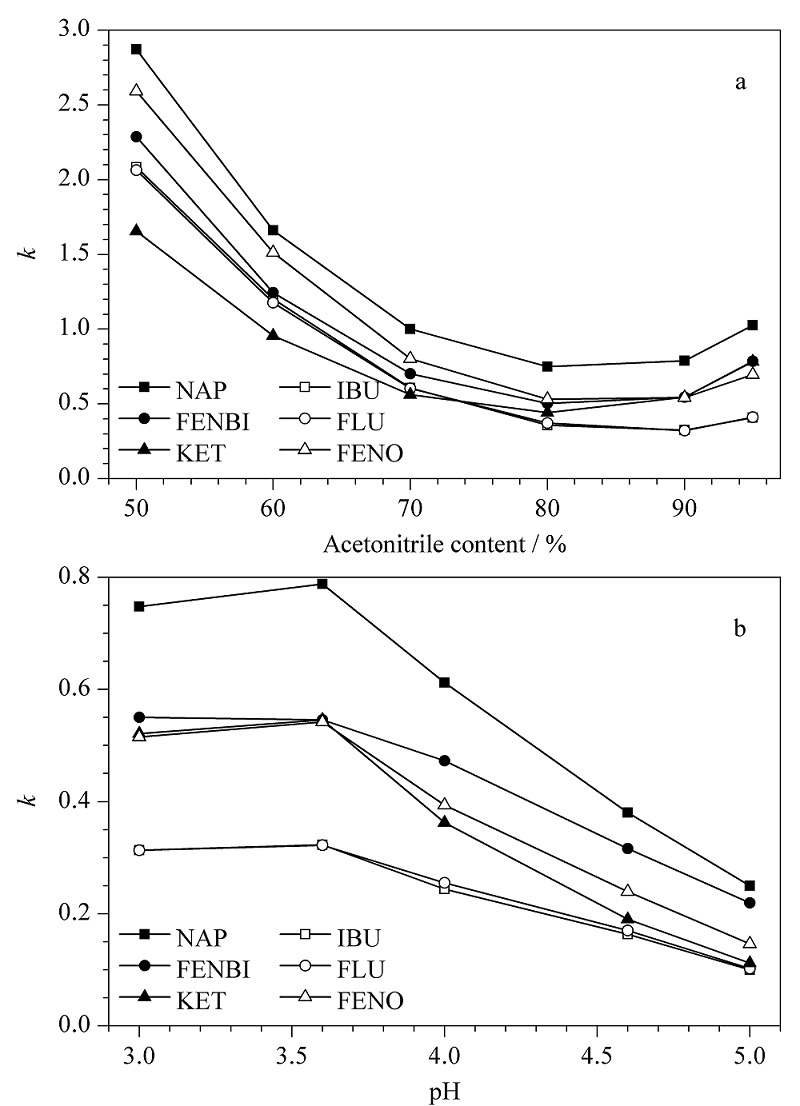
(a)流动相中乙腈含量和(b)流动相pH值对萘普生及其类似物在P1柱中色谱保留的影响

2.2.2 流动相pH值的影响

进一步研究了在液晶MIP上NAP及其类似物保留对pH的依赖性。从图3b可看出,在pH为3.6时,萘普生保留最大。尽管模板和类似物的保留都受到了流动相pH值的影响,但在pH 3.6~5.0时模板的保留因子变化趋势最为明显,因此pH对模板分子影响最大。MIP对模板的保留主要受到NAP解离水平的影响,在pH 3.0~3.6时,模板主要成分子状态,易与功能单体形成较多的印迹复合物,此时MIP对模板的保留较强。在更低pH条件下,溶剂中有比较多的游离氢离子与模板结合,影响其与功能单体形成印迹复合物,所以当pH值从3.6减少到3.0时,保留因子稍有减小;而在pH 3.6~5.0时,流动相酸碱度已超过NAP的p*K*_a_,模板萘普生中的-COOH成离子状态,不易形成氢键,印迹复合物的形成也受影响,所以NAP的保留因子也减少。

### 2.3 前沿分析

对突破曲线数据进行前沿分析,可求得MIPs的结合位点总数*B*_t_和解离常数*K*_d_(见表4)。在液晶MIPs中,虽然交联度15%时其印迹因子最大,但从前沿分析结果可以看到其结合位点总数并不是最大的。随着交联度从15%降低至7.5%, MIPs的结合位点数逐渐增大,但印迹因子减小,这可能是由于低交联度导致非特异性结合位点增加所致。相比于P3柱,交联度为26%的P1柱的结合位点总数较小,从而导致印迹因子较小;但是当交联度为20%和70%时,结合位点总数较大,而印迹因子却下降了。为了探究此处印迹因子下降的原因,进一步深入分析液晶MIPs识别的机理及规律,我们进行了以下的分析。

**表4:** 不同印迹整体柱的前沿分析结果及Langmuir、Freundlich拟合参数

Monolith	Frontal analysis				Langmuir fitting				Freundlich fitting			
	B_t_/μmol	K_d_/(mmol/L)	R		Q_0_/(mmol/L)	K_L_/(L/mmol)	R		K_F_/(mmol/L)	1/n		R
P1 (26%)	91.91	4.6	0.999		129.2	0.168	0.999		118.0	0.965		0.999
P2 (20%)	232.6	12.3	0.999		115.5	0.066	0.999		111.2	0.985		0.999
P3 (15%)	129.5	6.6	0.999		257.4	0.063	0.999		246.7	0.922		0.999
P4 (10%)	598.8	32.3	0.999		188.4	0.042	0.999		184.5	0.994		0.999
P5 (7.5%)	446.0	240.0	0.999		159.2	0.011	0.999		160.1	0.998		0.999
P7 (70%)	235.8	12.4	0.999		255.3	0.034	0.999		248.5	0.986		0.999

*B*_t_: number of binding sites; *K*_d_: dissociation constant; *R*: coefficient of determination; *Q*_0_: maximum adsorption capacity according to Langmuir monolayer adsorption; *K*_L_: constant according to the Langmuir model; *K*_F_: Freundlich constant related to adsorption capacity; 1/*n*: adsorption intensity of the adsorbent.

### 2.4 吸附等温线分析

2.4.1 Langmuir拟合

采用Langmuir模型对吸附等温线拟合可得各柱单层最大吸附量*Q*_0_和吸附系数*K*_L_(见表4)。从表4可以看出,交联度为15%时,*Q*_0_最大,这与前沿分析的*B*_t_值并不相符,但该结果与其具有最大的印迹效果是一致的,这表明Langmuir模型似乎更能科学地表征基于液晶单体的印迹系统的吸附性能。当交联度增大或者减小时,*Q*_0_均减小。此外,虽然交联度15%时的*Q*_0_与交联度70%时的相差不大,但其在柱效低于P7整体柱的情况下,也能展现出更加优异的印迹效果。从表4可以看出P3的印迹因子显著高于P7,这表明液晶单体在提高MIPs识别性能方面具有巨大潜力。

2.4.2 Freundlich拟合

采用Freundlich模型对吸附等温线拟合得到的数据见表4, *K*_F_越大表明吸附容量越高,1/*n*值越小表明非均一性能越强。从表4中可以看出,P3柱1/*n*最小,*K*_F_明显大于其他液晶印迹柱,表明P3具有较强的非均一性能和较高的吸附容量。交联度增大或者减小,其吸附容量(除P7外)和吸附能力都是减小,这与印迹因子的变化规律也是一致的。与Langmuir模型拟合结果类似,虽然P3的吸附容量稍低于P7,但其非均一性能要明显高于70%交联度的非液晶柱。另外,1/*n*值都小于1说明本实验制备的表面接枝印迹整体柱都是单层吸附^[13]^。

2.4.3 Scatchard分析

在本研究中,根据Scatchard方程发现*Q/C*对*Q*明显呈非线性关系,但曲线的两端分别有较好的直线关系,表明制备的液晶印迹柱具有两类结合位点:高亲和力位点和低亲和力位点。由Scatchard图两端的直线的斜率和截距可求得两类结合位点的平衡离解常数(*K*_d_)和最大表观吸附量(*Q*_max_)(见表5)。

**表5:** 不同印迹整体柱的Scatchard拟合参数

MIP	High affinity sites				Low affinity sites		
	Q_max_/ (mmol/ L)	K_d_/ (mmol/ L)	R		Q_max_/ (mmol/ L)	K_d_/ (mmol/ L)	R
P1(26%)	473	3.6	0.999		1117	8.8	0.992
P2(20%)	782	6.7	0.992		1370	11.8	0.906
P3(15%)	1366	4.6	0.999		1240	5.2	0.999
P4(10%)	2543	10.8	0.975		2661	13.5	0.947
P5(7.5%)	2329	14.5	0.971		9231	57.5	0.947
P7(70%)	1762	6.8	0.986		9169	36.0	0.911

*Q*_max_: maximum adsorption amount; *K*_d_: dissociation constant.

由表5可看出,只有交联度为15%时,高亲和位点的*Q*_max_高于低亲和位点的*Q*_max_,表明MIP的特异性吸附强于非特异性吸附。此外其他的MIP无论交联度增加或减少,高亲和位点的*Q*_max_始终低于低亲和位点的*Q*_max_,即MIP上特异性吸附量始终小于非特异性吸附量。例如,当交联度增加为26%时,MIP特异性结合位点减少,高亲和位点的*Q*_max_降低;当交联度减少至7.5%时,由于物理交联增大导致的非特异性结合位点增多,低亲和位点的*Q*_max_显著增加。对于交联度70%的非液晶印迹柱,其低亲和位点的*Q*_max_远高于高亲和位点的*Q*_max_,即其非特异性吸附远强于特异性吸附,这也解释了为什么前沿分析中P7的*B*_t_大于P3,而印迹因子却显著小于P3。但与前沿分析结果不同的是,交联度为20%的P2柱用Scatchard拟合的结合位点数要低于P3,这与印迹因子的变化规律是一致的,表明Scatchard分析也能准确直观地反映液晶印迹整体柱的识别特性。此外,交联度15%时的MIP解离常数也比较小,表明P3对于模板的结合力更强。

### 2.5 计量置换研究

在分子印迹聚合物的分子识别过程中,起主导作用的是三维孔穴结构还是模板分子与功能单体之间的相互作用,目前还存在分歧^[15]^。计量置换模型是在液相色谱体系中全面考察溶质、溶剂及固定相分子之间的各种相互作用和不同种类溶剂分子在固定相表面上的竞争吸附的理论模型^[26]^,因此被用来进一步分析液晶MIP的分子识别机制。

计量置换研究结果如表6所示,相关系数的绝对值都超过0.9,这证实了SDM理论可以成功地应用于基于液晶单体的分子印迹系统。在液晶印迹柱P1上,模板NAP的ln *A*值明显高于其类似物,说明P1对印迹分子具有更高的亲和性,即模板与功能单体的非共价相互作用。在印迹柱上,当不同的溶质分子被吸附在固定相上时,如果印迹分子的结构越匹配MIPs的空腔结构,印迹分子覆盖的活性位点数量越高,从固定相释放的相应数量溶剂分子也就越高,即*nβ*越高^[15]^。从表6可以知道,印迹柱上的空穴结构与模板NAP匹配性很高(*nβ*=36.3),但稍次于其类似物酮洛芬(*nβ*=39.5)。但P1对空间最匹配的酮洛芬的亲和力(ln *A*=0.242)不及模板分子(ln *A*=0.645)的1/2,这说明空间效应不是决定该液晶印迹系统的分子识别能力的主要因素。

**表6:** 液晶印迹整体柱P1的SDM-R结果

Analyte	ln A	nβ	r
NAP	0.645	36.3	-0.975
IBU	-0.389	28.3	-0.974
FLU	-0.365	28.9	-0.963
KET	0.242	39.5	-0.983
FENBI	0.304	30.6	-0.973
FENO	0.123	30.9	-0.966

ln *A*: total affinity potential between the solute and stationary phase; *n*: number of strong solvent molecules released from the stationary phase; *β*: constant related to solvents; *r*: correlation coefficient.

### 2.6 分子识别热力学

根据Van’t Hoff公式,以ln *k*和ln *α*分别对1/*T*作图,得到了直线关系图(见图4)。在试验温度范围内,模板和3个类似物的ln *k*和ln *α*的Van’t Hoff线性拟合良好,并且保留因子随着温度升高而减少。表7总结了NAP及其类似物在不同交联度MIPs上的焓变、熵变和焓变差、熵变差。对于低交联液晶MIPs柱,即26%、15%、7.5%交联柱,|ΔΔ*H*|<*T*|ΔΔ*S*|,这表明低交联液晶印迹系统的分离过程是一个熵控制的过程。而对于高交联非液晶MIPs柱,即70%交联度柱,|ΔΔ*H*|>*T*|ΔΔ*S*|,则表明该非液晶MIPs的分离过程是一个焓控制的过程。

**图4:**
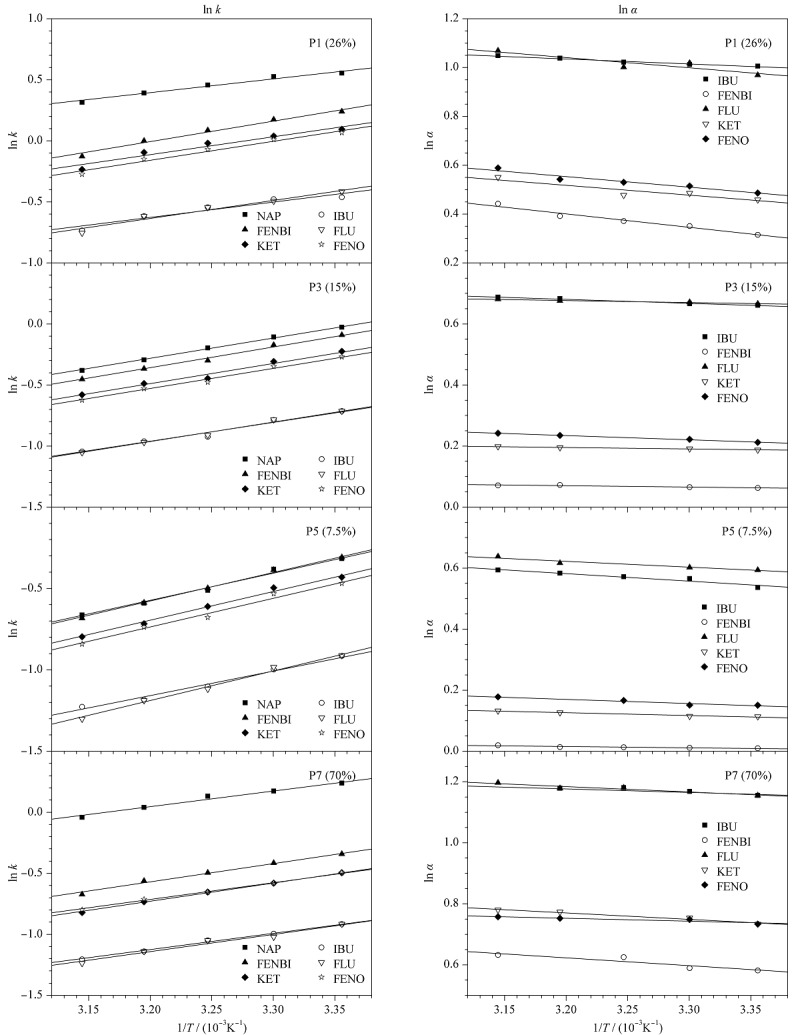
萘普生与其类似物在不同交联度整体柱上的保留因子以及分离因子(*α*)随温度变化的Van’t Hoff图

**表7:** 不同交联度的印迹整体柱的热力学参数

Monolith	Analyte	ΔH/(kJ/mol)	ΔS/(J/(mol·K))	r_1_	ΔΔH/(kJ/mol)	ΔΔS/(J/(mol·K))	r_2_
P1 (26%)	NAP	-9.60	-27.5	0.987			
	IBU	-10.7	-39.4	0.962	1.75	14.2	-0.988
	FENBI	-14.3	-45.7	0.990	4.67	18.3	-0.983
	FLU	-12.6	-45.6	0.979	3.55	20.0	-0.916
	KET	-12.5	-40.9	0.974	3.44	15.3	-0.931
	FENO	-13.3	-43.8	0.988	3.68	16.4	-0.971
P3 (15%)	NAP	-14.1	-47.5	0.999			
	IBU	-13.2	-50.2	0.989	1.11	9.23	-0.995
	FENBI	-14.5	-49.4	0.996	0.385	1.82	-0.953
	FLU	-13.7	-51.7	0.994	0.554	7.40	-0.981
	KET	-14.1	-49.1	0.991	0.421	2.97	-0.987
	FENO	-14.0	-49.3	0.994	1.17	5.70	-0.998
P5 (7.5%)	NAP	-14.2	-50.3	0.995			
	IBU	-13.5	-50.8	0.993	2.11	11.6	0.966
	FENBI	-15.0	-52.7	0.999	0.358	1.27	-0.929
	FLU	-15.5	-59.6	0.995	1.63	10.4	-0.967
	KET	-15.0	-53.8	0.996	0.801	3.61	-0.983
	FENO	-15.0	-54.1	0.993	1.16	5.14	-0.964
P7 (70%)	NAP	-10.9	-34.6	0.991			
	IBU	-11.4	-45.7	0.998	12.9	14.0	-0.919
	FENBI	-12.7	-45.5	0.995	12.2	12.1	-0.923
	FLU	-12.0	-47.8	0.987	15.1	14.7	-0.956
	KET	-12.7	-46.8	0.999	17.9	12.7	-0.983
	FENO	-11.7	-43.4	0.999	8.32	8.92	-0.918

Δ*H*: enthalpy changes; Δ*S*: entropy changes; ΔΔ*H*: difference in enthalpy changes; ΔΔ*S*: difference in entropy changes. *r*_1_ and *r*_2_ represent the correlation coefficients of ln *k* and ln *α* to 1/*T*, respectively (Equations (7) and (8)).

## 3 结论

本文制备了一系列低交联度液晶MIPs,通过与常规的高交联度MIPs比较分析发现,液晶的加入具有显著提高吸附特异性和吸附容量的能力。热力学研究可以看到低交联度液晶印迹整体柱展现出了与高交联度非液晶印迹整体柱不同的保留机制。总之,本文对低交联液晶印迹柱的保留机制和识别热力学的研究为新一代MIP固定相的特性提供了更为深入地认识,未来我们将对该类色谱固定相传质机理进行深入研究,为液晶MIP这种新型固定相的理性设计及合成提供有益的信息。
